# Syntrophy emerges spontaneously in complex metabolic systems

**DOI:** 10.1371/journal.pcbi.1007169

**Published:** 2019-07-24

**Authors:** Eric Libby, Laurent Hébert-Dufresne, Sayed-Rzgar Hosseini, Andreas Wagner

**Affiliations:** 1 Integrated Science Lab, Umeå University, Umeå, Sweden; 2 Department of Mathematics and Mathematical Statistics, Umeå University, Umeå, Sweden; 3 Santa Fe Institute, Santa Fe, New Mexico, United States of America; 4 Department of Computer Science, University of Vermont, Burlington, Vermont, United States of America; 5 Department of Evolutionary Biology and Environmental Studies, University of Zurich, Zurich, Switzerland; University of Illinois at Urbana-Champaign, UNITED STATES

## Abstract

Syntrophy allows a microbial community as a whole to survive in an environment, even though individual microbes cannot. The metabolic interdependence typical of syntrophy is thought to arise from the accumulation of degenerative mutations during the sustained co-evolution of initially self-sufficient organisms. An alternative and underexplored possibility is that syntrophy can emerge spontaneously in communities of organisms that did not co-evolve. Here, we study this *de novo* origin of syntrophy using experimentally validated computational techniques to predict an organism’s viability from its metabolic reactions. We show that pairs of metabolisms that are randomly sampled from a large space of possible metabolism and viable on specific primary carbon sources often become viable on new carbon sources by exchanging metabolites. The same biochemical reactions that are required for viability on primary carbon sources also confer viability on novel carbon sources. Our observations highlight a new and important avenue for the emergence of metabolic adaptations and novel ecological interactions.

## Author summary

By exchanging resources, the members of a microbial community can survive in environments where individual species cannot. Despite the abundance of such syntrophy, little is known about its evolutionary origin. The predominant hypothesis is that syntrophy arises when originally independent organisms in the same community become interdependent by accumulating mutations. In this view, syntrophy arises when organisms co-evolve. In sharp contrast we find that different metabolism can interact syntrophically without a shared evolutionary history. We show that syntrophy is an inherent and emergent property of the complex chemical reaction networks that constitute metabolism.

## Introduction

Syntrophy is a frequent property of microbial communities. It allows a community as a whole to survive in an environment, even though individual members cannot [1–6]. For example, methanogenic archaea remove the hydrogen waste of fermenting bacteria, which helps both partners survive in low energy environments [7]. The mutual dependence between different organisms shared by all syntrophies is thought to originate in a process of sustained co-evolution. In this process, ancestrally self-sufficient organism are driven to interdependence by degenerative mutations that erode their metabolic independence [8–11]. An alternative but underexplored possibility is that syntrophy can emerge spontaneously from serendipitous combinations of organisms with complementary biochemical abilities. If so, syntrophy does not require a shared evolutionary history and is not a degenerative phenomenon. Here, we study this *de novo* origin of syntrophy in the networks of biochemical reactions that constitute metabolism.

While different kinds of ecological interactions are called syntrophic, most involve a mutual nutritional dependency that is mediated through an exchange or transfer of molecules such as nutrients or waste products [1, 12]. In the case of fermenting bacteria and methanogenic archaea, the bacteria generate hydrogen waste that can inhibit their ability to ferment, thereby preventing growth [13]. By consuming the hydrogen, the archaea not only grow but they also enable the continued growth of the bacteria. Many other syntrophies exhibit a similar producer/consumer dynamic mediated by waste molecules, and a favorable coupling of biochemical reactions [14–16]. Some forms of syntrophy involve a two-way exchange of molecules. For example, pairs of microbes engineered to be deficient in essential amino acids, can survive by exchanging the missing amino acids when co-cultured [17, 18]. In all cases of syntrophy, a central outcome is that a community of organisms can survive in environments where individuals cannot.

Since syntrophy entails a mutual dependency between organisms, it raises the basic evolutionary question of how such dependencies originate [9, 19]. The canonical view is that they originate from the accumulation of degenerative mutations during sustained co-evolution between initially self-sufficient organisms [8–10, 20]. However, a series of experimental studies [8, 19] observed the early evolution of a syntrophy using a co-culture of two organisms with no known history of interaction, but whose metabolisms complement each other through the exchange of molecules. Another experimental study [21] found that the yeast *Saccharomyces cerevisiae* and the alga *Chlamydomonas reinhardtii* can evolve a dependent cross-feeding relationship in environments without access to carbon dioxide. Moreover, other combinations of ascomycetous yeasts and Chlamydomonas species can have such relationships. Similarly, computational metabolic modeling approaches [22–24] have identified pairings of well-known bacterial species with metabolic complementarity facilitated by the exchange of metabolites in particular environments.

While studies like these show how syntrophy may emerge in pairs of metabolically complementary organisms, they do not identify the origins of the complementarity nor do they exclude the possibility that metabolic complementarity is a result of co-evolution. Even if we could prove that two organisms shared *none* of their evolutionary history, their metabolic complementarity might be a byproduct of co-evolution with other organisms—which is difficult to disprove. The question then is whether syntrophy is the result of an evolutionary process or if it is a natural consequence of coupling metabolisms. To address this question, it is important to systematically eliminate the influence of evolutionary history. Thus, we use a computational approach to evaluate the potential for syntrophy in metabolic networks that have no evolutionary history.

## Results

**Fig 1:**
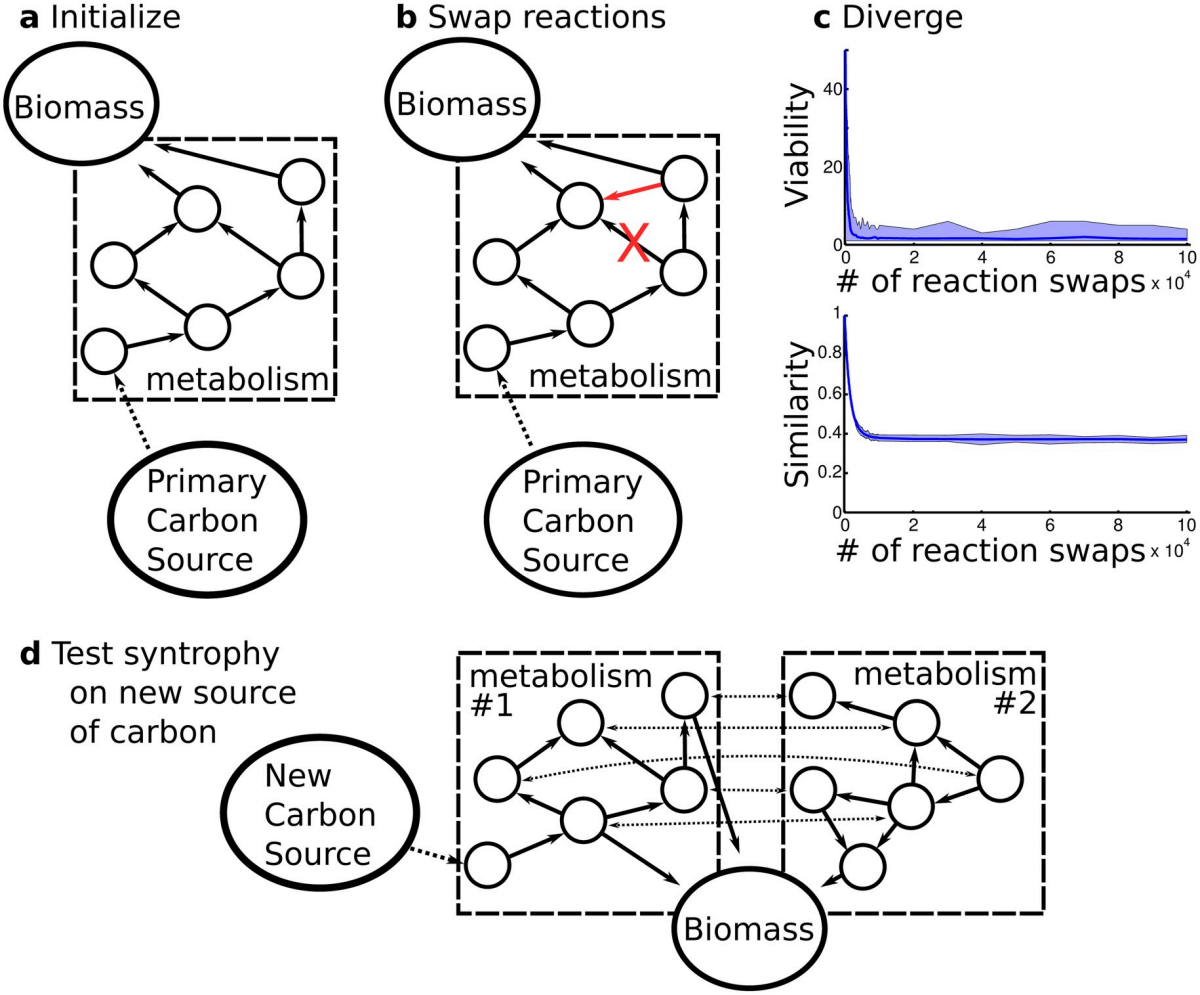
Outline of the computational framework shows method to generate metabolisms without evolutionary history. a) We start with an initial metabolic network that is viable on a primary carbon source. b) We delete a reaction from a network and add a new reaction from the universe of reactions, where each such reaction swap must maintain viability on the primary carbon source. c) Through thousands of such reaction swaps, metabolic networks diverge from each other, lose viability on non-primary carbon sources, and become randomized within the constraints of viability (see [32] and Fig S1 and Fig S2 in S1 Appendix for more details). d) We evaluate syntrophy by allowing random viable metabolisms to exchange molecules and assessing viability on a new carbon source. In parts of our analysis where the identity of exhcnaged molecules is unimportant, we can increase computational efficiency by pooling reactions from two metabolisms.

The starting point for our work is a curated model of *E. coli* metabolism [25], which is a network of biochemical reactions that comprises 2,079 reactions. We focus on 50 carbon sources on which *E. coli* is viable (Table S1), i.e., its metabolism can produce all essential biomass molecules in a minimal medium containing one of these 50 carbon sources as the only carbon source [26, 27]. Because we aim to identify generic properties of metabolic systems rather than properties of *E. coli* metabolism, we need to create an ensemble of metabolisms that (i) harbor the same number of reactions as *E. coli*, (ii) are viable on at least one of the specific carbon sources that *E. coli* is also viable on, but (iii) contain an otherwise random complement of reactions drawn from the known “universe” of biochemical reactions. To produce such an ensemble of metabolic networks, we use an established Markov Chain Monte Carlo sampling technique [25] (see Fig 1 for a schematic overview of the approach). Briefly, in this technique the *E. coli* metabolic network is altered in a step-wise fashion, where each step deletes a single reaction and adds another reaction chosen at random from the reaction universe. We then use flux balance analysis [28], a computational technique whose predictions are in good agreement with experimental data [29–31], to predict the metabolism’s viability in a focal environment containing a single, *primary* carbon source. If the altered metabolism is viable, the change is accepted, otherwise it is reverted. By repeating this procedure 50,000 times, one essentially performs a random walk through the space of all possible metabolisms that are viable on the primary carbon source. During this random walk a metabolism preserves its viability on the primary carbon source but its complement of reactions becomes essentially randomized, diverging rapidly from the *E. coli* ancestor (see Fig S1 in S1 Appendix). It loses viability on most of the initial 50 carbon sources, and ultimately remains viable on only the primary carbon source and potentially a few additional carbon sources (see Fig S2 in S1 Appendix).

**Fig 2:**
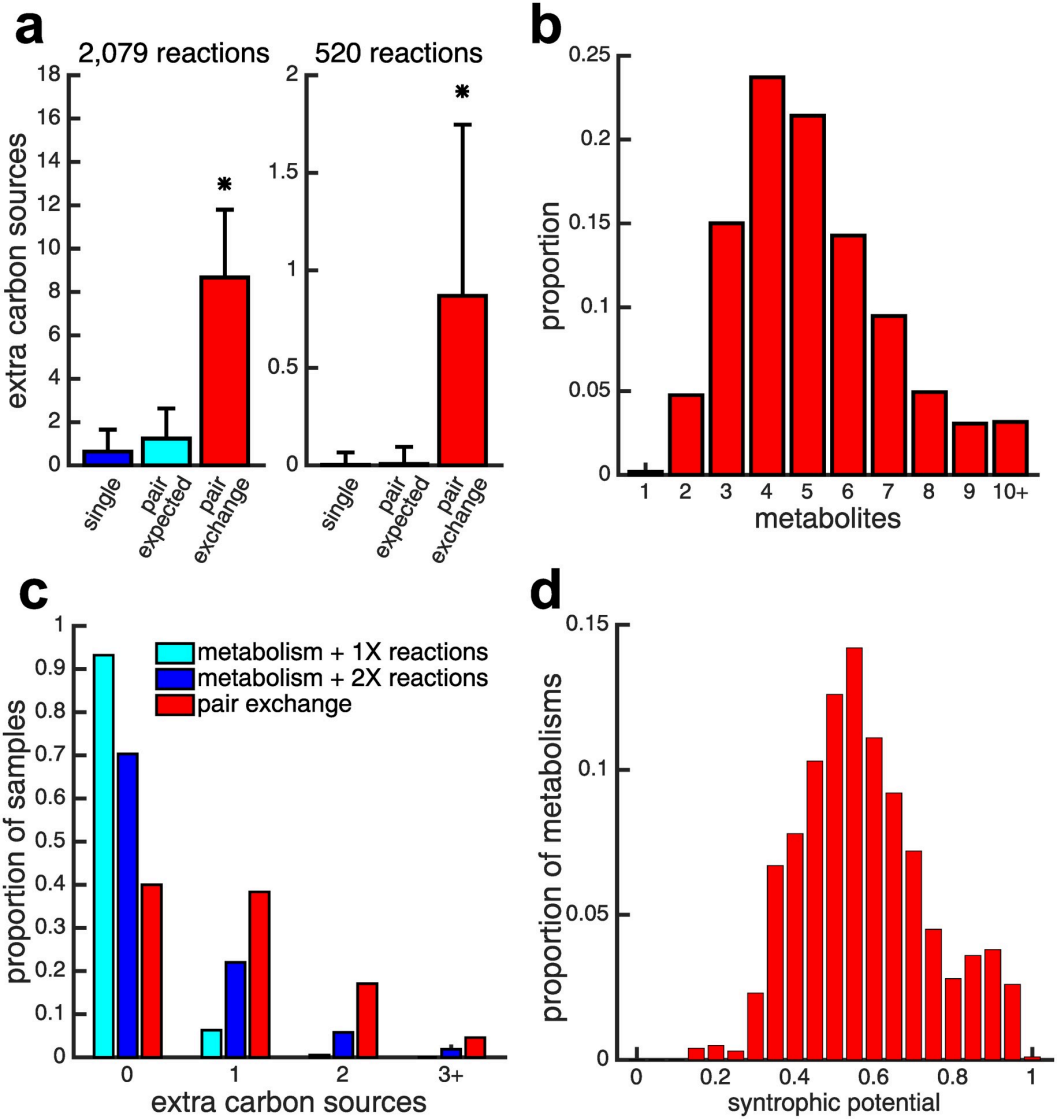
Syntrophy emerges frequently when pairs of random viable metabolisms interact. a) The average number of additional carbon sources that metabolisms with 2,079 (left) or 520 (right) reactions are viable on is shown for single metabolisms (blue), for pairs of metabolisms expected by chance alone (cyan), and for pairs of metabolisms in our data (red). Error bars indicate the standard deviation. We observe significantly more syntrophy than expected by chance alone (asterisk, *p* < 10^−10^, sign test). b) Shown is the distribution of the minimum number of metabolites that must be exchanged for a syntrophic interaction, for pairs of size-reduced metabolisms composed of 520 reactions. c) Distributions of the number of additional carbon sources that can be metabolized via syntrophy are shown for pairs of size-reduced metabolisms (red) as well as random single metabolisms augmented by 520 (cyan) and 1,040 (blue) randomly chosen reactions. Augmented single metabolisms are viable on fewer additional carbon sources than pairs of interacting metabolisms. d) The distribution of syntrophic potential for 1,000 random size-reduced metabolisms ranges from ≈.15 to 1, indicating that every metabolism can produce a syntrophic interaction in at least 15% of pairings.

With this sampling method, we create samples of 20 random viable metabolisms for each of the 50 primary carbon sources, resulting in 1,000 random viable metabolisms in total, each with 2,079 reactions. A collection of random viable metabolisms like this allows us to quantify the likelihood that syntrophy emerges when two metabolisms can exchange metabolites. To this end, we allow pairs of metabolisms to exchange metabolites and determine their viability on carbon sources that neither member of a pair could utilize in isolation (see Methods). For all 499,500 unique pairs of the 1,000 sampled metabolisms, we compute the number of carbon sources on which each pair is viable. In addition to the primary carbon source, the average individual metabolism is viable on only 0.64 ± 1.02 additional carbon sources. In the absence of syntrophy, we would thus expect that a pair of metabolisms is viable on roughly twice that many additional carbon sources (1.24 ± 1.39, see Methods). Instead, we find that pairs of metabolisms are viable, on average, on 13.6 times more novel environments, i.e., on 8.67 ± 3.13 additional carbon sources beyond what they are individually viable on (see Fig 2A), which is significantly greater (*p* < 10^−10^, sign test).

Our random viable metabolisms harbor many more reactions than are actually needed to grow on any one carbon source. On average, each metabolism has only 365.65 ± 10.32 reactions (≈17.6% of the *E. coli* reaction network) that when removed preclude viability on its primary carbon source. It is possible that the overabundance of excess reactions is solely responsible for the observed extent of syntrophy. To evaluate this possibility, we generate another set of 1, 000 metabolisms—20 for each of the 50 primary carbon sources—but reduce the size of the metabolisms to a quarter of the original size, 520 reactions. Although 520 is still above the absolute smallest size for growth on a single primary carbon source, it is dramatically (75%) smaller than that of *E. coli*.

Not surprisingly, the number of additional carbon sources on which individual reduced metabolisms are viable is smaller than for *E. coli*-size metabolisms. Only four out of the 1,000 reduced metabolisms can grow on a carbon source other than their primary carbon source, and these four metabolisms can grow on only a single additional carbon source. Without syntrophy, we would thus expect that a pair of metabolisms is viable on 0.008 ± 0.09 additional carbon sources. However, pairs of these size-reduced metabolisms are actually able to grow on 0.87 ± 0.88 novel carbon sources on which neither metabolism could grow in isolation (see Fig 2A). This number is more than 100 times greater than expected by chance, a difference that is statistically highly significant (*p* < 10^−10^, sign test). In addition, the number of transferred metabolites required for syntrophy is small. On average only 5 metabolites need to be exchanged between size-reduced metabolisms to grow on a novel carbon source (see Fig 2B). In larger, *E. coli*-sized networks this number of metabolites is 3.5 on average, and thus somewhat smaller (see Fig S3 in S1 Appendix).

By exchanging metabolites, pairs of metabolisms effectively pool their reactions and form a larger joint metabolism. Perhaps the frequent syntrophy we observe is strictly due to the larger number of reactions in a joint metabolism, and does not require any integration of these reactions into a functional metabolic network. To assess this possibility, we augment each size-reduced metabolism with a set of unique reactions that are randomly sampled from the known reaction universe and equivalent in number to another size-reduced metabolism. (We note that each reaction from the reaction universe is present in at least four of our size-reduced metabolisms.) We find that only 6.8% of such augmented metabolisms can grow on an additional carbon source, compared to 60% of pairs of size-reduced metabolisms that exchange metabolites. Even if we augment size-reduced metabolisms with a number of reactions equivalent to two size-reduced metabolisms, we still find that pairs of functional metabolisms generate syntrophy significantly more often—both in terms of frequency and the number of additional carbon sources (see Fig 2C, significance assessed by chi-squared test).

It is possible that a small subset of metabolisms are responsible for most of the syntrophic interactions we observe. To evaluate this possibility, we define the syntrophic potential of a metabolism as the fraction of its pairings with other metabolisms that produce a syntrophic interaction. Fig 2D shows that the distribution of syntrophic potentials of our sampled metabolisms has a median of 0.58, meaning that an average sampled metabolism can interact syntrophically with 58% of other metabolisms. All sampled metabolisms can produce syntrophic interactions with at least 15% of other metabolisms, and we find a similar prevalence of syntrophy using a complementary method for sampling metabolisms that does not begin with an *E. coli* metabolism (see Fig S5 in S1 Appendix). Syntrophy is not just a property of a small subset of metabolisms.

**Fig 3:**
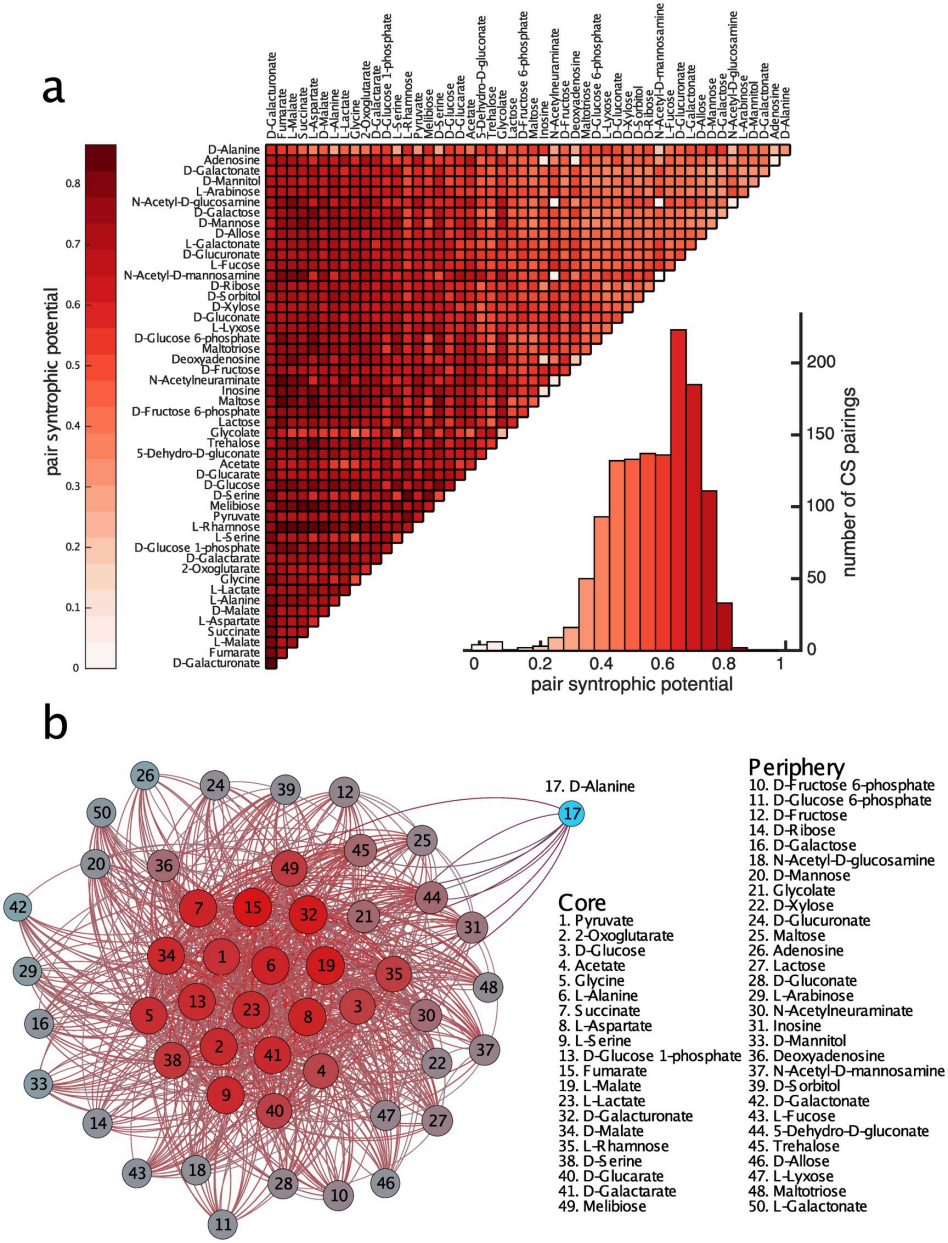
Primary carbon sources affect the syntrophic potential of metabolisms. a) The triangular grid shows the carbon source pair syntrophic potential, i.e. the fraction of pairs of metabolisms viable on a given pair of primary carbon sources that produce a syntrophy, for all pairs of primary carbon sources. The histogram summarizes the data and shows that different pairs of carbon sources vary widely in their syntrophic potential. b) Network representation of the data in a) where nodes are carbon sources and edges are weighted by the pair syntrophic potential between nodes (only edges above a threshold weight of .55 are shown for clarity). The network structure is disassortative, with a core of carbon sources (red) that have high pair syntrophic potential with one another and a periphery (gray) whose carbon sources have low pair syntrophic potential with each other but high pair syntrophic potential with the core. D-Alanine (cyan) is the sole outlier to this structure.

The likelihood of a syntrophic interaction may also depend on the primary carbon sources on which the interacting metabolisms are viable. To explore this dependency, we consider all 1,275 combinations of two primary carbon sources, and define the “carbon source pair syntrophic potential” as the incidence of syntrophy when pairs of metabolisms viable on these primary carbon sources interact. Fig 3a shows this carbon source pair syntrophic potential in a triangular grid for all pairs of carbon sources. We include in this analysis metabolisms viable on the same primary carbon source. The data shows a broad distribution, with some primary carbon source pairs having especially high syntrophic potential. For example, 86.5% of pairs of metabolisms viable on primary carbon sources L-Rhamnose and L-Aspartate interact syntrophically. In contrast, pairs of metabolisms each viable on N-Acetyl-D-mannosamine never do. The pair syntrophic potential we compute are significantly correlated with potentials derived from a complementary sampling method that does not begin with an E. coli metabolism (see Fig S6 in S1 Appendix). The distribution of the carbon source pair syntrophic potentials has a larger variance than expected by chance alone (see Fig S7 in S1 Appendix). In sum, primary carbon sources play an important role for the emergence of syntrophic interactions.

To better understand the effect of the primary carbon source on the probability that a pair of metabolisms interacts syntrophically, we construct a network in which nodes are carbon sources and edges are weighted by the corresponding carbon source pair syntrophic potential. We find that this network has a core-periphery, or disassortative, structure. The core is composed of a set of 20 carbon sources that have high pair syntrophic potential with each other. In contrast, the periphery is composed of 29 carbon sources that have low pair syntrophic potential with each other but higher pair syntrophic potential with the core. The one outlier to the core/periphery distinction is D-Alanine (see Fig S8 and S9 in S1 Appendix). In general, the disassortative network structure implies that metabolisms viable on some primary carbon sources (the core) are more likely to generate syntrophy than metabolisms viable on others (the periphery). A similar disassortative network structure exists for *E. coli*-sized metabolisms (see Fig S10 in S1 Appendix).

**Fig 4:**
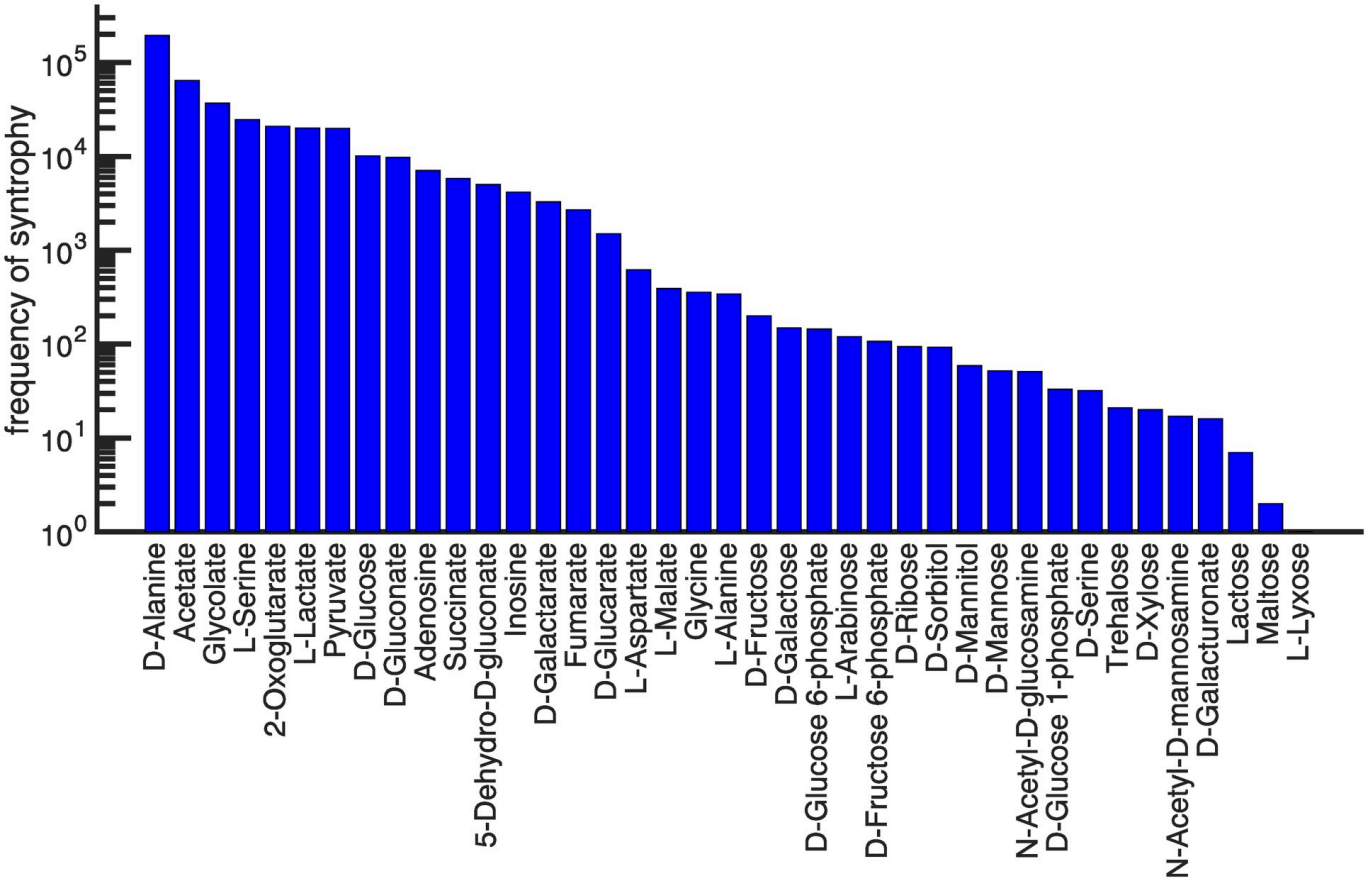
Different carbon sources vary widely in how readily they are metabolized syntrophically. The frequency at which carbon sources can be metabolized syntrophically is shown for 39 primary carbon sources. The frequency ranges from one instance for L-Lyxose to 194,576 instances for D-Alanine. The remaining 11 carbon sources (not shown) were never metabolized syntrophically.

We now turn our attention to the novel carbon source environments that become accessible through syntrophy. Of the 50 carbon sources, 39 can be metabolized through syntrophic interactions by at least one pair of metabolisms. The incidence of syntrophy, however, varies by five orders of magnitude across carbon sources (see Fig 4). At one extreme is L-Lyxose which can be metabolized syntrophically by only one pair of metabolisms. At the other extreme is D-Alanine, which can be metabolized syntrophically by 194,576 pairs of metabolisms. It accounts for 44.83% of all observed syntrophic interactions. This incidence of syntrophy is three times greater than the next most frequent carbon source syntrophy, acetate (14.88%).

**Fig 5:**
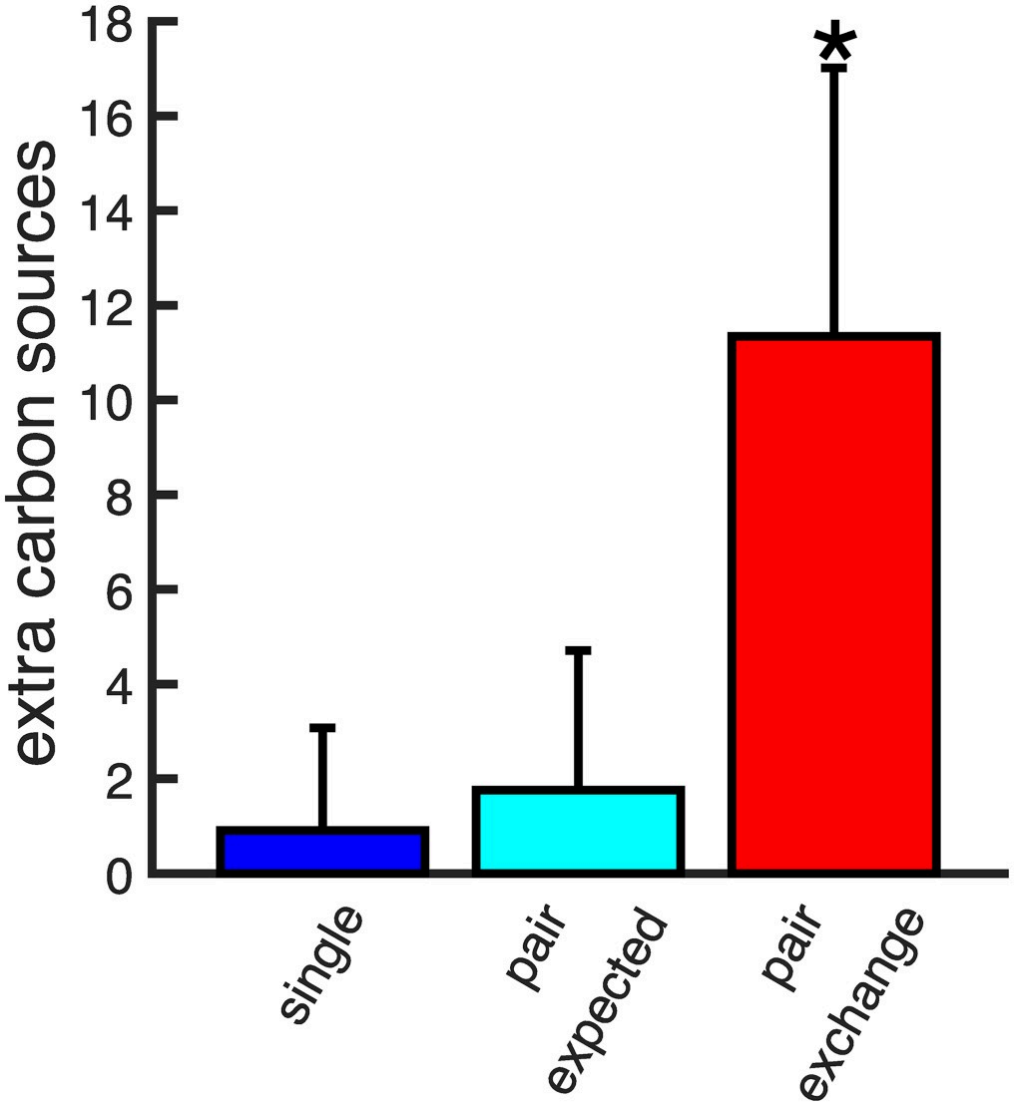
Syntrophy increases significantly in size-reduced metabolisms when transport of all primary carbon sources is guaranteed. Shown is the average number of additional carbon sources that metabolisms with 520 reactions are viable on when carbon transport is restored, for single metabolisms (blue), for pairs of metabolisms expected by chance alone (cyan), and for pairs of metabolisms in our data (red). Error bars indicate the standard deviation. We observe significantly more syntrophy when pairs of metabolisms can exchange metabolites than expected by chance alone (asterisk, *p* < 10^−10^, sign test).

In order to use a nutrient, a metabolism needs to be able to transport it into the cell. This ability, which usually requires specific transport proteins, is represented by transport “reactions” in computational models of metabolism [22]. In sampling random metabolisms, we allow these transport reactions to be gained and lost just like other reactions, and find that the inability to transport primary carbon sources is often the only obstacle to syntrophy (see Fig S11 and Fig S12 and analyses in S1 Appendix). For example, when we restore transport of all primary carbon sources, syntrophy becomes 13 times more frequent than we have reported so far. That is, with restored transport of primary carbon sources, size-reduced metabolisms can grow on 11.34 additional carbon sources compared to 0.87 when transport may be lost (see Fig 5).

**Fig 6:**
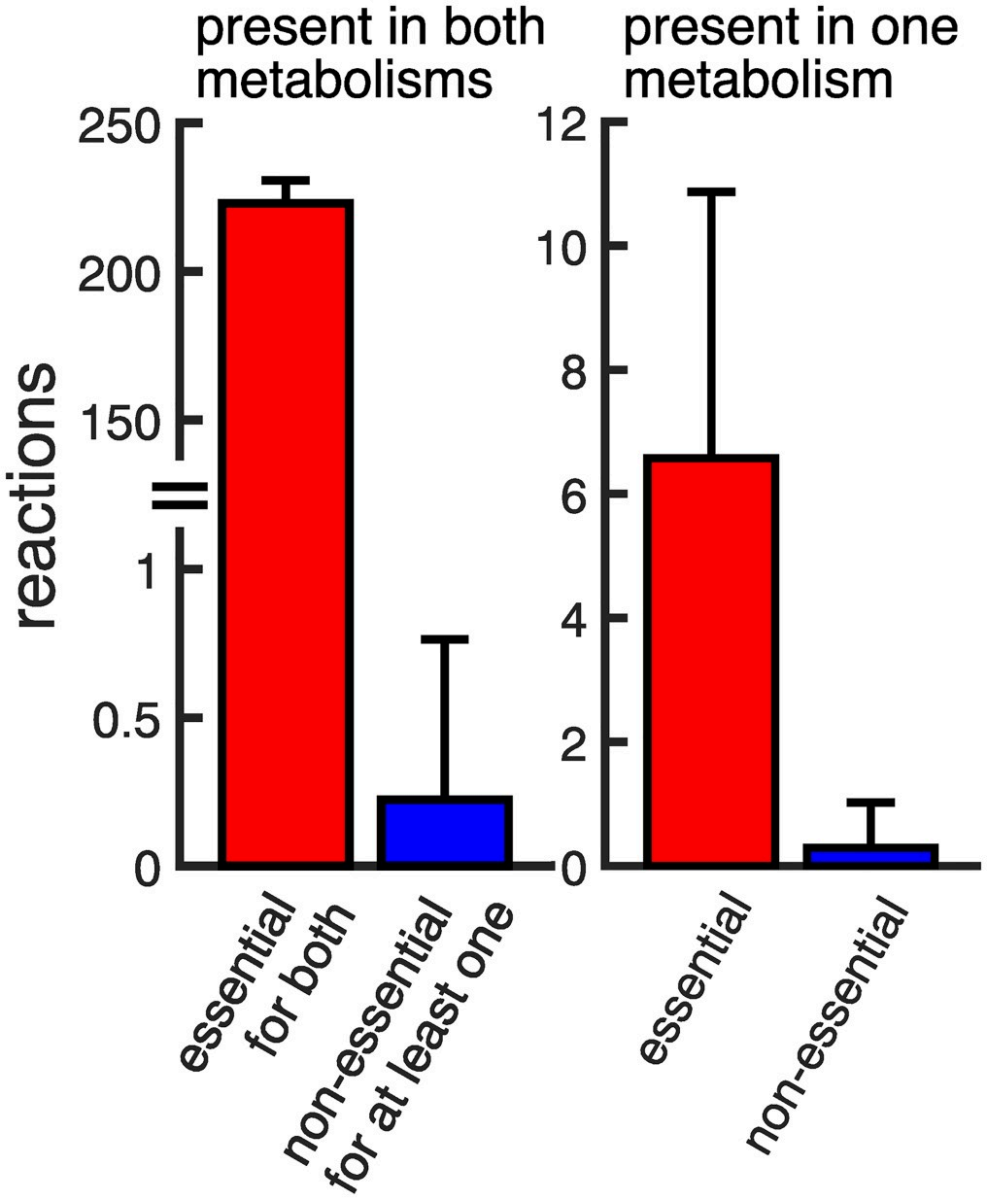
Most essential reactions are also essential for growth on a primary carbon source. For those reactions that are essential for a syntrophic interaction and found in both metabolisms, the vast majority (over 99%) are also essential for each metabolism to grow on its primary carbon source when in isolation. Reactions present in only a single metabolism are more often essential than non-essential for growth on a primary carbon source (sign-test, *p* < 10^−10^). Bars indicate means of 100,000 randomly sampled syntrophies and error bars indicate a single standard deviation.

Although transport into cells is necessary for viability on a new carbon source, it is only a first step that needs to be followed by the ability to metabolize the carbon source. We next ask about the reactions necessary to do so and why they are present in pairs of random metabolisms. To this end, we first identified those reactions that are essential for a syntrophic interaction. These are reactions whose removal prevents viability on the new carbon source (see Methods). We find that of the 785 unique reactions that exist on average in a pair of size-reduced metabolisms, 230 reactions (29%) are essential for the syntrophy between these metabolisms. Further analysis shows that 99.87% of these reactions are also essential for at least one of the metabolisms to be viable on its primary carbon source (see Fig 6). Thus, a metabolism that is required to be viable on one carbon source harbors a complement of reactions necessary for syntrophy with another metabolism. In other words, syntrophy can emerge when viable metabolisms come to inhabit the same environment.

## Discussion

Here, we assess a route to syntrophy that does not rely on co-evolution. We use a computational approach to obtain metabolic reaction networks that have no evolutionary history but are capable of converting a primary carbon and energy source into all essential biomass molecules. We show that syntrophy emerges frequently when such metabolisms exchange molecules. The biochemical reactions that facilitate survival on new carbon sources through syntrophy are also those necessary for growth on the original, primary carbon sources. Thus, syntrophy does not depend on a set of dispensable reactions that are useful only in specific environmental and ecological contexts. Such reactions would become quickly eliminated through deleterious mutations without selection for their maintenance. Instead, syntrophy emerges as a by-product of the ability to metabolize a primary carbon source and does not require a shared evolutionary history.

Our study sheds light on the evolutionary origins of obligate mutualisms in microbes. The vast majority of microbes cannot be cultured in the laboratory, one reason being that such microbes depend on metabolic exchange with other microorganisms to synthesize essential biomass molecules [33, 34]. Theoretical explanations of such obligate mutualisms usually assume that the mutualism is a derived (evolved) state, or that it resulted from a serendipitous event in which microbes with complementary abilities came to coexist in the same habitat. The first assumption has received more attention both theoretically and experimentally, particularly in regards to obligate host-endosymbiont relationships and the Black Queen hypothesis [8, 9, 20, 35–37]. The second assumption has received less attention, even though syntrophy or cross-feeding readily emerges when complementary organisms coexist in specific environments [38–41]. A possible reason for this neglect is that the likelihood of *de novo* metabolic complementarity is difficult to evaluate.

Prior computational studies that investigated metabolic complementarity used models of specific organisms [22–24, 40] which have been shaped by their evolutionary history. In contrast, our paper focuses on the biochemical structure found in metabolism itself. Our observation that syntrophy emerges frequently when metabolic networks interact suggests that any potential challenge to establishing an obligate mutualism does not lie in metabolic complementarity. Instead it lies in ecological or environmental factors that determine which molecules can be exchanged [33].

A caveat to our approach is the assumption that metabolisms can freely exchange metabolites, because limitations on such exchange could constrain syntrophy. We did not impose any such limitations, because they depend on the chemical environment, ecological factors, and physiological conditions [33, 42, 43]. For example, when the endosymbiotic bacterium *Buchnera aphidicola* is grown outside of its aphid host experimentally, it secretes different metabolites depending on the chemical resources in its environment [42]. Experimentally characterized syntrophies differ widely in the number and types of molecules exchanged, from a single waste molecule between methanogenic archaea and fermenting bacteria [7], to a handful of amino acids in engineered bacterial populations [18], to 58 metabolites between *Buchnera aphidicola* and its host [44]. However, since the number of exchanged metabolites is itself subject to evolutionary change, imposing limits based on observations in extant organisms requires specific assumptions about a shared eco-evolutionary history that we wanted to avoid. That being said, we found that syntrophy required the exchange of only a modest number (2-5) of metabolites in our networks, which have fewer reactions than *E. coli*. Other computational studies that also assumed free metabolite exchange yield predictions that are consistent with experimental data [22, 31, 45].

Multiple ecological factors determine whether or not syntrophy actually emerges [35], even where two organisms have complementary metabolic abilities. For example, whether organisms will actually exchange molecules depends on the energetic costs to produce them [23, 37, 40, 46, 47]. If energetically costly metabolites are made available to other organisms in the environment, interspecies competition or the evolution of non-cooperating ‘cheater’ genotypes can prevent syntrophic exchange [48–50]. In contrast, if such metabolites can be directly transferred between organisms, or if they are waste products without energetic value to their producer, opportunities for cooperative interactions increase [20, 23, 35, 38, 51, 52]. However, even compounds with identical cost and availability can lead to more than one kind of ecological relationship. For instance, depending on the molecular stability and toxicity of an excreted waste compound, either exploitation, competition, or mutualism can evolve betweeen microbes [53]. For these reasons, no one study like ours can identify all sufficient conditions for the spontaneous emergence of syntrophy. Rather, our work determines that the most important necessary condition—metabolic complementarity—is easily met.

Finally, our work reveals yet another avenue for the non-adaptive origins of complex traits, an origin whose potential importance was first recognized by Darwin [25, 54–58]. Since syntrophy is an emergent property of metabolic networks, it can act as a potential driving force in the eco-evolutionary dynamics of natural communities. For example, by allowing a community to use new carbon sources, syntrophy can improve community survival in the face of shifts in resource availability [59]. Alternatively, it can facilitate colonization of new environments by nascent, obligate mutualisms. Thus, while co-evolution is not strictly necessary for the origins of syntrophy it can provide an opportunity for the evolution of more complex community interactions.

## Materials and methods

### Sampling metabolisms

We implement an evolutionary algorithm that generates metabolisms viable on specific, primary carbon sources but contain an otherwise random complement of biochemical reactions. It starts with an initial *E. coli* metabolism that is viable on 50 carbon sources, where viability is assessed using flux balance analysis and a biomass growth function defined in [26, 27]. A metabolism is considered viable on a carbon source if for 10 concentration units of that carbon source, the biomass growth function exceeds.001 product yield of all compounds (see Fig S4 in S1 Appendix for justification). We choose one out of these 50 possible carbon sources, and call it the primary carbon source.

We then use an iterative Markov Chain Monte Carlo (MCMC) algorithm that swaps reactions between the metabolism and a curated list of 6,588 possible biochemical reactions (a “universe” of reactions) based on the LIGAND database of the Kyoto Encyclopedia of Genes and Genomes used in [25]. In each step of the MCMC algorithm, a reaction is randomly chosen to be eliminated, and a replacement reaction, chosen at random from the reaction universe, is inserted into the metabolism. If the resulting metabolism is still viable on the primary carbon source, we call the swap successful and use it as a starting point for a second swap. If, instead, the metabolism is no longer viable on the primary carbon source, we reject the swap and revert to the metabolism before the swap. We continue this process for 50,000 steps, a number sufficiently large to effectively randomize the reaction complement [32] (see Fig S1 and Fig S2 in S1 Appendix). To create a population of 1,000 metabolisms, we run the algorithm 20 times for each of the 50 primary carbon sources. For each primary carbon source, between 83.9% and 84.4% of reaction swaps were successful.

Previous work on random viable metabolisms revealed that even when they are required to grow only on a single carbon source, they can often grow on several additional carbon sources [25]. For our analysis, it was important to keep this number of additional carbon sources small, in order to better assess metabolic innovations caused by syntrophy. To this end, we did not limit which reactions could be gained/lost in the MCMC sampling—with the exception of the biomass reaction used to assess viability. Consequently, transport reactions which allow carbon sources to be brought into the cell could be lost, rendering metabolisms nonviable on those carbon sources. We explore the effects of allowing transport of all metabolites in the Supplementary material.

To generate the 1,000 reduced metabolisms with 520 reactions, we start with each of the 1,000 *E. coli*-sized metabolisms, and randomly remove reactions that are not essential for growth on the primary carbon source, because the flux through them is equal to zero. Alternatively, we could reduce the original *E. coli* metabolism to 520 reactions and then iterate the MCMC algorithm for 50,000 MCMC steps. Doing so, however, significantly reduces the acceptance rate of reaction swaps from 84% to 31% and thus increases computational cost, because metabolic networks become more constrained due to their small size and higher proportion of essential reactions. Ultimately, our approach produces greater diversity among sampled metabolisms. For example, the average number of shared reactions between two size-reduced metabolisms generated by our approach is ≈270 compared to ≈282 using the MCMC algorithm on an initially size-reduced metabolism.

### Exchange and evaluation of syntrophy

To evaluate whether a pair of metabolisms can produce a syntrophic interaction, we allow the free exchange of all metabolites. In practice, we might expect there to be physical constraints limiting the movement of some metabolites but this would depend on the particular environmental context and may be even be ameliorated through evolution. Instead, we consider a form of metabolite exchange equivalent to models of leaky exchange [60, 61]. For a syntrophic interaction, a pair of metabolisms must be able to generate all essential biomass molecules in an environment that contains a specific carbon source. We determine whether this is possible by pooling the unique reactions of a pair of metabolisms and constructing a new joint metabolism. We then use flux balance analysis to compute the biomass growth flux of the pooled pair on carbon sources that neither of the two individual metabolisms are viable on in isolation. We note that this computational procedure is mathematically equivalent to solving a more explicit model in which metabolisms are kept distinct and a set of exchange reactions are added that permit biochemical compounds to be transferred from one metabolism to another. However, our procedure reduces the computational cost of the linear programming problem solved in the flux balance analysis. We do, however, use the explicit exchange reactions in our analyses where we determine the minimum number of metabolites required for a syntrophy, following the methodology described in [22].

### Formal definition of syntrophy and syntrophic potential

We denote a random viable metabolism that is viable on primary carbon source *C*_*i*_ as *M*_*i*,*x*_ where *i* ∈ [1, 50] indexes the carbon source, and *x* ∈ [1, 20] indexes the individual random viable metabolism. We use *G*(*M*_*i*,*x*_, *M*_*j*,*y*_, *C*_*k*_) to represent the growth rate of a pair of metabolisms *M*_*i*,*x*_ and *M*_*j*,*y*_ on carbon source *C*_*k*_. The growth rate of a single metabolism, *M*_*i*,*x*_, on a carbon source, *C*_*k*_, can be represented as *G*(*M*_*i*,*x*_, *M*_*i*,*x*_, *C*_*k*_). Combining these two notations, metabolisms *M*_*i*,*x*_ and *M*_*j*,*y*_ interact syntrophically if for some *k* the following holds *G*(*M*_*i*,*x*_, *M*_*i*,*x*_, *C*_*k*_) = 0, *G*(*M*_*j*,*y*_, *M*_*j*,*y*_, *C*_*k*_) = 0, and *G*(*M*_*i*,*x*_, *M*_*j*,*y*_, *C*_*k*_) > 0. We note that for numerical computations we consider growth rates below a tolerance of .001 to be equivalent to 0. If two metabolisms interact syntrophically then we say that *S*(*M*_*i*,*x*_, *M*_*j*,*y*_) = 1 and if they do not then *S*(*M*_*i*,*x*_, *M*_*j*,*y*_) = 0, where *S* is a function that indicates the presence of a syntrophy. We note that by definition no metabolism can produce a syntrophy with itself, i.e. *S*(*M*_*i*,*x*_, *M*_*i*,*x*_) = 0. Using this notation, we can express the syntrophic potential of a metabolism $\bar{s}\left(M_{i,x}\right)$, i.e., the fraction of pairings involving a particular metabolism *M*_*i*,*x*_ that results in a syntrophy, as $\bar{s}\left(M_{i,x}\right)=\langle S\left(M_{i,x},M_{j,y}\right)\rangle =\sum_{j=1}^{50}\sum_{y=1}^{20}S\left(M_{i,x},M_{j,y}\right)/999$, where 999 is the number of unique pairs of metabolisms involving a particular *M*_*i*,*x*_. We also define a similar metric called the (carbon source) pair syntrophic potential $\bar{s_{p}}\left(C_{i},C_{j}\right)$, which quantifies the fraction of pairings between metabolisms viable on primary carbon sources *C*_*i*_ and *C*_*j*_ that result in a syntrophy. It computes as $\bar{s_{p}}\left(C_{i},C_{j}\right)=\sum_{x=1}^{20}\sum_{y=1}^{20}S\left(M_{i,x},M_{j,y}\right)/400$ when *i* ≠ *j* and $\bar{s_{p}}\left(C_{i},C_{i}\right)=\sum_{x=1}^{20}\sum_{y=x+1}^{20}S\left(M_{i,x},M_{i,y}\right)/190$ when *i* = *j*. The 400 and 190 terms correspond to the number of unique pairs of metabolisms given that there are 20 sampled metabolisms per primary carbon source. We note that $\bar{s_{p}}\left(C_{i},C_{j}\right)=\bar{s_{p}}\left(C_{j},C_{i}\right)$.

### Assessing the value of metabolic exchange

To determine if the extent of syntrophy observed in pairings of random metabolic networks is more than what we would expect by chance, we construct a simple null model in which we assume that there is no exchange of intermediate metabolites. In this case, the probability that a pair is viable on a carbon source is simply the probability that at least one of the constituent metabolisms is individually viable on it. If a metabolism required to be viable on carbon source *C*_*i*_ has a probability *p*_*i*_ of being viable on some additional carbon source *C*_*k*_ (*k* ≠ *i*), then the probability that the pair of metabolisms, whose members are required to be viable on *C*_*i*_ and *C*_*j*_, is also viable on a particular additional carbon source is 1 − (1 − *p*_*i*_)(1 − *p*_*j*_). The expected number of additional carbon sources the pair is viable on is then given by (50 − 2)(1−(1 − *p*_*i*_)(1 − *p*_*j*_)) if the primary carbon sources of the metabolisms are different and (50 − 1)(1 − (1 − *p*_*i*_)(1 − *p*_*j*_)) if they are the same. We compute this expectation for all pairs of metabolisms. As an estimate for the probability *p*_*i*_, we use the number of carbon sources a metabolism is viable on beyond its primary carbon source.

## Supporting information

S1 Appendix Supporting information. Additional analyses and figures to support the results of the paper.
(PDF)
