# Supplementary material for: Syntrophy emerges spontaneously in complex metabolic systems
Source: PLoS Comput Biol. 2019 Jul 24;15(7):e1007169. doi: 10.1371/journal.pcbi.1007169 (PMC6655585; doi:10.1371/journal.pcbi.1007169)
Supplement: S1 Appendix — Additional analyses and figures to support the results of the paper. (PDF) [file pcbi.1007169.s001.pdf]

Supplementary material: Syntrophy emerges spontaneously in complex metabolic systems

Eric Libby[1,2], Laurent Hébert-Dufresne[2,3], Sayed-Rzgar Hosseini[4], and Andreas Wagner[2,4]

[1]Umeå University, Umeå, Sweden
[2]Santa Fe Institute, Santa Fe, NM, USA
[3]University of Vermont, Burlington, VT, USA
[4]University of Zurich, Zurich, Switzerland

Primary carbon sources

Carbon sources (1-25)	Carbon sources (26-50)
1. Pyruvate	26. Adenosine
2. 2-Oxoglutarate	27. Lactose
3. D-Glucose	28. D-Gluconate
4. Acetate	29. L-Arabinose
5. Glycine	30. N-Acetylneuraminate
6. L-Alanine	31. Inosine
7. Succinate	32. D-Galacturonate
8. L-Aspartate	33. D-Mannitol
9. L-Serine	34. D-Malate
10. D-Fructose 6-phosphate	35. L-Rhamnose
11. D-Glucose 6-phosphate	36. Deoxyadenosine
12. D-Fructose	37. N-Acetyl-D-mannosamine
13. D-Glucose 1-phosphate	38. D-Serine
14. D-Ribose	39. D-Sorbitol
15. Fumarate	40. D-Glucarate
16. D-Galactose	41. D-Galactarate
17. D-Alanine	42. D-Galactonate
18. N-Acetyl-D-glucosamine	43. L-Fucose
19. L-Malate	44. 5-Dehydro-D-gluconate
20. D-Mannose	45. Trehalose
21. Glycolate	46. D-Allose
22. D-Xylose	47. L-Lyxose
23. L-Lactate	48. Maltotriose
24. D-Glucuronate	49. Melibiose
25. Maltose	50. L-Galactonate

Table S1: **List of primary carbon sources.** This is the list of the 50 primary carbon sources that we use in our analyses. It is a subset of the carbon sources on which *E. coli* can grow when each of these carbon sources is provided as the sole carbon source in a minimal growth medium.

Additional data for *E. coli*-sized metabolisms

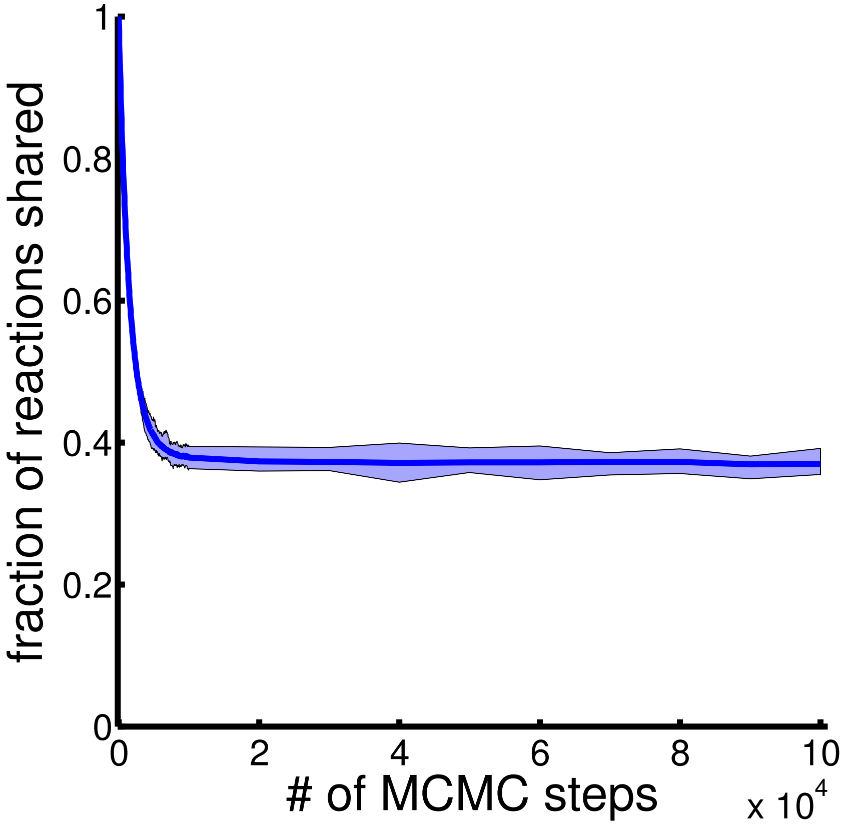

Figure S1: **Divergence in reaction similarity with ancestor over the course of MCMC sampling of metabolism.** For each carbon source C in the set of 50 carbon sources in Table S1 we perform MCMC sampling, where each step of the sampling requires viability on carbon source C. At regular intervals during the sampling, we compare the set of metabolic reactions of the metabolism undergoing the MCMC random walk to the ancestor, i.e., to the metabolism at the start of the MCMC sampling. Plotted is the proportion of reactions that are identical between the MCMC strain and the ancestor (mean over 50 MCMC runs in dark blue; range from minimum to maximum in light blue). We find that at 50,000 steps of the MCMC sampling the fraction of shared reactions is 0.37 ± 0.01 which is not significantly different from this fraction at 100,000 MCMC steps (also 0.37 ± 0.01, $P = 0.56$; Wilcoxon sign-rank test).

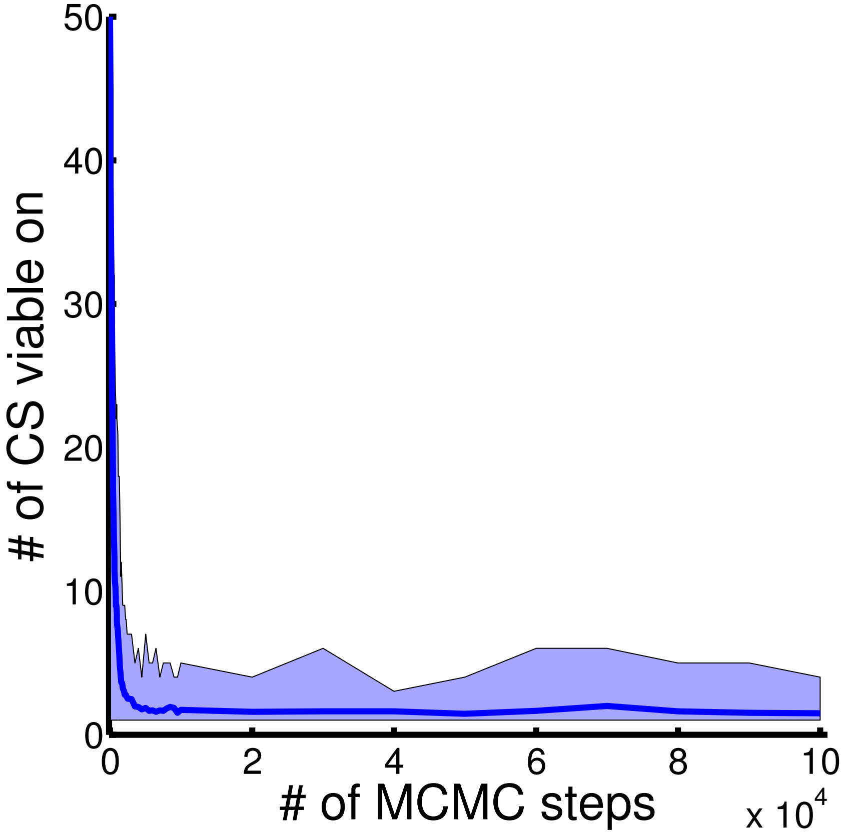

Figure S2: **Loss of carbon source viability during MCMC sampling.** For each carbon source C in the set of 50 carbon sources in Table S1 we perform MCMC sampling, where each step of the sampling requires viability on carbon source C. At regular intervals during the sampling, we determine how many carbon sources the metabolism undergoing the MCMC random walk is viable on. Plotted in the graph is the mean (dark blue) and range from minimum to maximum (light blue) of the number of carbon sources. We find that metabolisms quickly lose viability on carbon sources beyond C. By 50,000 MCMC steps, metabolisms are not viable on significantly more carbon sources than at 100,000 MCMC steps (1.44 ± 0.81 versus 1.48 ± 0.79, $p \approx 0.80$, Wilcoxon sign-rank test).

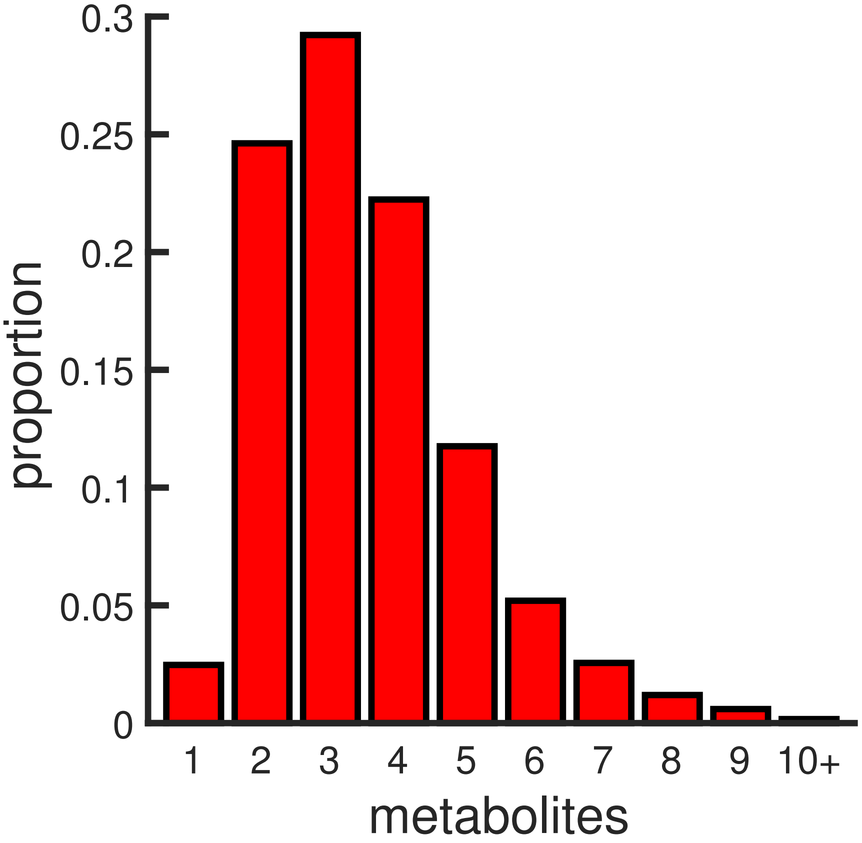

Figure S3: **The minimum number of transferred metabolites for a syntrophic interaction between *E. coli*-sized metabolisms.** Shown is the distribution of the minimum number of metabolites that must be transferred to enable a syntrophic interaction in a random sample of 1,000 syntrophically interacting pairs of metabolisms. This is a companion plot to Figure 1B but for larger metabolic networks. The average number of transferred metabolites is ≈ 3.5, which is smaller than for the size-reduced networks (average of 5 metabolites). Moreover, 90% of sampled syntrophies require five or fewer metabolites to be transferred.

Additional data for MCMC sampling algorithm

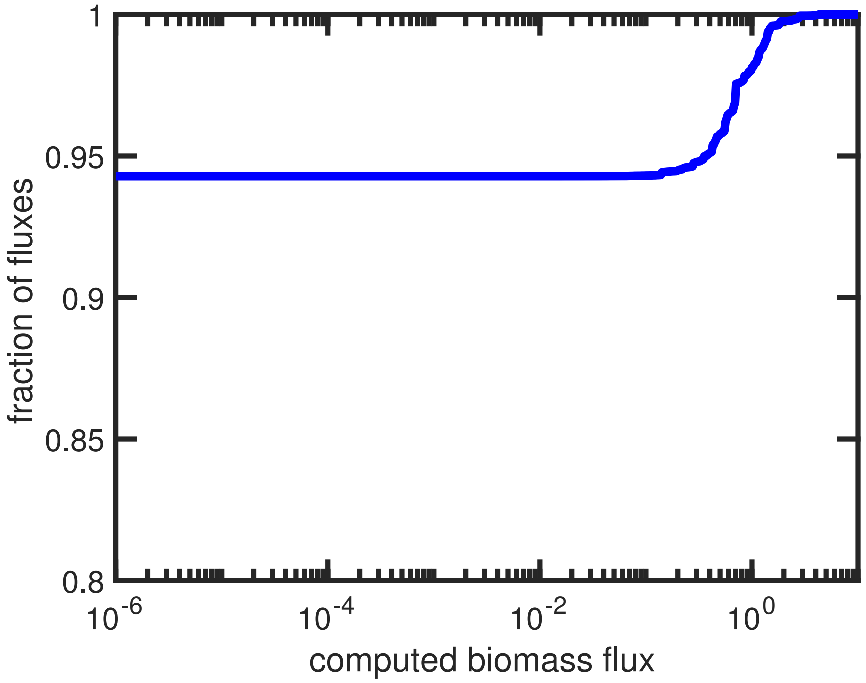

Figure S4: **An empirical cumulative distribution function plot shows the distribution of biomass yield computed for all pairings of size-reduced metabolisms.** Viability on a carbon source is assessed by using flux balance analysis to maximize the flux through the biomass reaction, which acts as a proxy for the growth rate of an organism. We use a cutoff of 10^{-3} flux to determine viability. The empirical cumulative distribution function plot shows the flux determined for all possible pairs of size-reduced metabolisms provided with 10 units of each carbon source. The great majority of flux rates (94.3%) are below 10^{-6}. There are no fluxes computed between 10^{-6} and 10^{-3} and the rest of the fluxes are between 10^{-3} and 5.

A complementary method for sampling metabolisms

We describe a method for generating random samples of metabolisms that is complementary to our MCMC algorithm. Instead of starting with an initial *E. coli* metabolism and swapping reactions, we begin with the set of all possible reactions in the reaction universe and randomly remove reactions one at a time while maintaining viability on a primary carbon source. We continue this pruning process until there are 520 reactions, which is the same number as in the size-reduced metabolisms in the main paper. Using this method, we generated 20 random metabolisms for each of the 50 carbon sources, i.e., a total of 1000 metabolisms. We determined the viability of each metabolism on additional carbon sources, and found that all of the metabolisms are only viable on their primary carbon source. We then considered all possible pairs of this new set of metabolisms and assessed their viability on additional carbon sources. We found that 64.8% of pairings resulted in a syntrophic interaction. Figure S5 shows the distribution of syntrophic potentials.

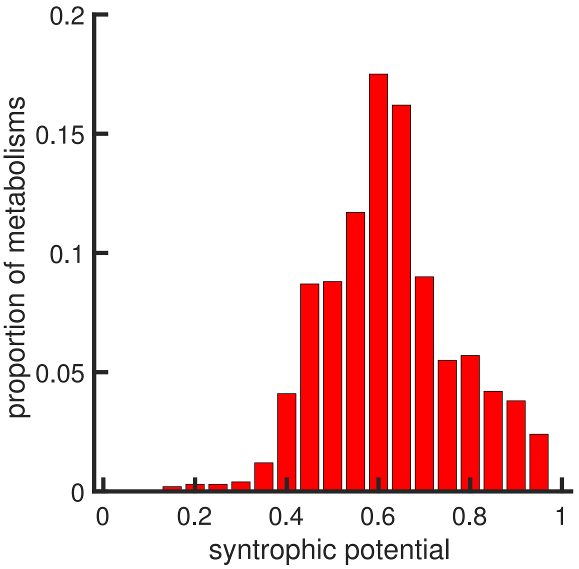

Figure S5: **The distribution of syntrophic potentials for 1,000 random size-reduced metabolisms sampled via pruning.** The plot is a complement to Figure 1d and shows that syntrophy is similarly frequent if we sample random metabolism via pruning. The syntrophic potentials we observe ranges from ≈ .15 to .997 which is almost identical to what we found with MCMC-sampled metabolisms (syntrophic potential between ≈ .15 and 1). Again, every metabolism can produce a syntrophic interaction in at least 15% of pairings, and a small set of metabolisms that can produce a syntrophic interaction with almost every other metabolism (996 out of 999).

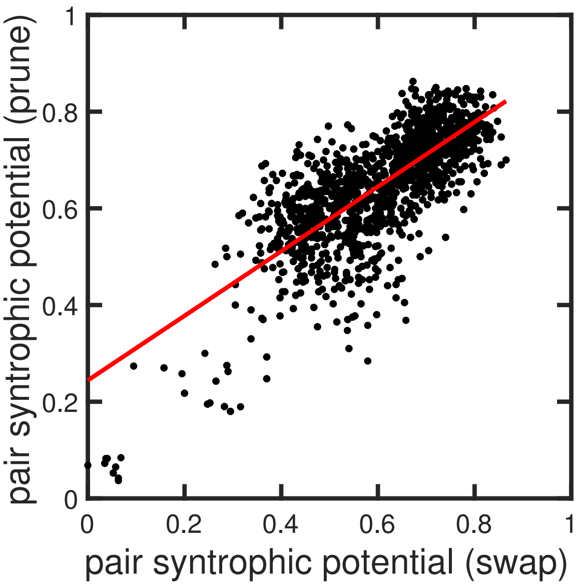

Figure S6: **Comparison of carbon source pair syntrophic potentials between two sets of sampled metabolisms.** Each black circle represents a pair syntrophic potential for two carbon sources using metabolisms sampled by reaction pruning (vertical axis) versus metabolisms sampled by reaction swapping (horizontal axis, data used in analyses in main paper). The correlation coefficient between the two sets of carbon source pair syntrophic potentials is .75, which is statistically significantly different from zero($p < 10^{-10}$). The red line shows the best fit line following a linear regression. (The slope is also statistically significantly different from zero with $p < 10^{-10}$.)

Significance of primary carbon source effects

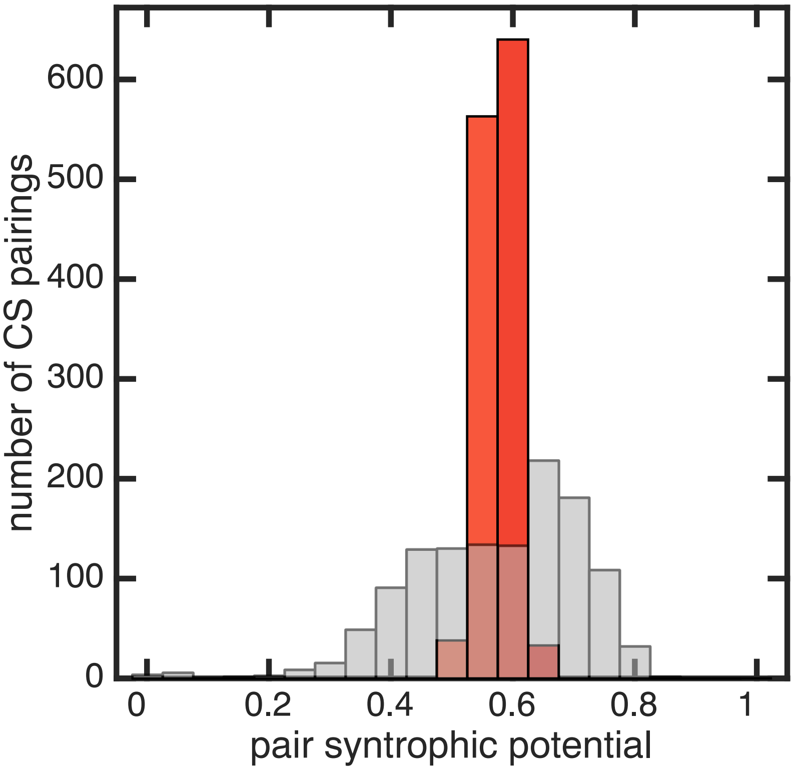

Figure S7: **Null distribution of syntrophic interactions between metabolisms selected for viability on particular carbon sources.** We randomly reassign metabolisms to carbon sources and compute the fraction of pairings between carbon sources that yield syntrophy, a quantity that we call the pair syntrophic potential. The result is a distribution of pair syntrophic potentials. We repeat this process 100 times and show the distribution with the highest variance (plotted in red). We find that this distribution has smaller variance compared to what is observed in the actual carbon source assignments (plotted in gray and also Figure 2a).

Network analysis

To analyze how the primary carbon sources affect the probability of two metabolisms producing a syntrophic interaction, we construct a network consisting of 50 nodes. Each node represents one carbon source. The nodes are connected by weighted, undirected edges with a weight corresponding to the pair syntrophic potential defined in the main text (see Fig. S8). We further define the syntrophic potential of a particular carbon source as the sum of its pair syntrophic potentials, i.e., the potential of carbon source i is $s_i = \sum_j \bar{s_p}(C_i, C_j)$. This potential provides a measure of the probability that a metabolism viable on that carbon source is likely to produce a syntrophy when paired with any other metabolism, regardless of the carbon source on which that other metabolism is viable.

The resulting network has a strong core-periphery structure such that carbon sources can be classified as belonging to either a core or a periphery. Whether a carbon source is in the core or in the periphery depends on the carbon sources that its metabolisms are more likely to produce syntrophy with, i.e., it depends on the syntrophic potential of the carbon sources with whom it "shares" most of its potential. In network terms, the network structure is highly disassortative: metabolism viable on carbon sources with low syntrophic potentials (a low sum of pair syntrophic potentials) produce most of their syntrophies when paired with metabolisms viable on carbons sources with high potential, more so than would be expected by chance alone, i.e., given random syntrophic pairings.

We quantify the disassortativity in our network by examining correlations between the potentials of primary carbon sources whose metabolisms show syntrophic interactions. For each primary carbon source C_i, we compute its average partner's syntrophic potential, which is simply an average of the pair syntrophic potentials of all carbon source pairs (C_i, C_j) that include i, weighted by how often the partner carbon source C_j is involved in syntrophic interactions with other carbon sources, i.e., by C_j's syntrophic potential. More specifically, for primary carbon source C_i, the average partner's potential equals $\sum_j \bar{s_p}(C_i, C_j) s_j / 50$. To illustrate this computation, consider the following hypothetical example. If 80 percent of metabolisms viable on carbon source i show syntrophies with metabolisms whose primary carbon sources have a high syntrophic potentials of 0.75, and 20 percent show syntrophies with metabolisms whose primary carbon sources have low syntrophic potentials of 0.25, then the resulting average partner's potential would calculate as 0.8(0.75)+0.2(0.25). Using this concept, our measure of dissasortativity is the correlation between the potential of each carbon source and its average partner's potential (see Fig. S9).

In a network with a core-periphery structure, we expect a negative correlation between the syntrophic potentials of two nodes. In other words, in a syntrophic interaction involving two specific nodes where one node belongs to the periphery, the other is likely to belong to the core. In contrast, in a network without a core-periphery structure, the pair syntrophic potential of two carbon sources is determined by their individual potentials, and without any correlations between these potentials—even two carbon sources with low potential may

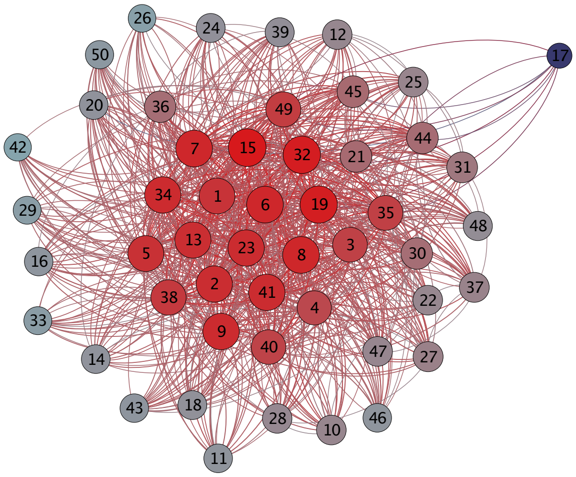

Figure S8: **Network of carbon sources and pair syntrophic potentials.** The network shows a strong core-periphery structure, which is evident from the observation that peripheral nodes have more connections to the core than each other. Nodes are colored according to their PageRank score[1] (red=high, gray=low), and sized according to their syntrophic potentials. Numbers correspond to the carbon sources as listed in Table S1. To avoid clutter, edges corresponding to a syntrophic potential below 0.55 are not shown.

sometimes be involved in syntrophic interactions. Fig. S9 shows that the carbon source network indeed has a core-periphery structure. The periphery consists of primary carbon sources whose metabolisms produce a majority of their syntrophic interactions – more than expected by chance alone – when paired with carbon sources of the core.

Because a syntrophic interaction is more likely to involve a carbon source with high syntrophic potential than one with a low potential, it is possible that the observed core-periphery structure is simply a consequence of the node potentials in a finite-sized network, and could thus occur by chance alone. To find out, we produced 100 random networks by shuffling which pairs of metabolisms produce syntrophies. In this reshuffling, every carbon source maintained its syntrophic potential, but the pair syntrophic potentials (edge weights) were redistributed randomly among all edges. We then again compared the potential of a carbon source with its average partner's potential in both the real data and the rewired networks, see Fig. S9. In the real data, the core-periphery manifests itself as a correlation: peripheral carbon sources interact syntrophically mostly with core carbon sources, and thus have average partners with a higher than expected potential. However, the correlation is lost in the rewired networks. This confirms that the core-periphery observed in our data

[1] Page rank score algorithm implemented according to: Newman, M. Networks: an introduction. (Oxford university press, 2010).

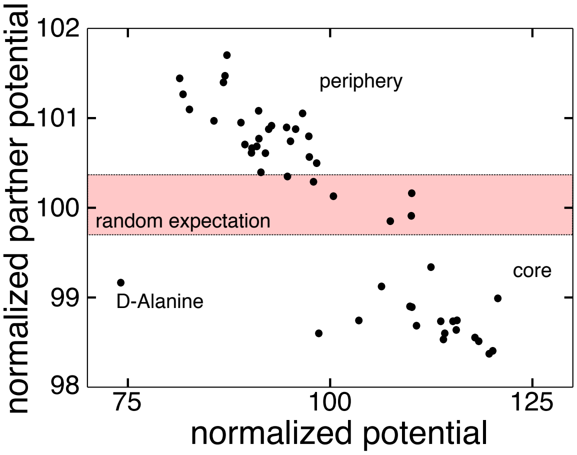

Figure S9: **Correlation of syntrophic potentials** The core-periphery structure of the carbon source network from Figure S8 mostly stems from the correlation between the syntrophic potential of a carbon source (network node) and the average syntrophic potential of its partners (weighted by the carbon source pair syntrophic potentials). Potentials are displayed as a percentage of the average to highlight which primary carbon sources produce more or fewer syntrophic interactions on average. The linear correlation coefficient is -0.74 in the real data, and vanishes in the rewired networks (shown in the red area which corresponds to a 95% confidence interval for data from 100 randomized networks).

is statistically significant, as our data lie well outside of the fluctuations observed in the rewired networks.

The direct consequence of this core-periphery structure is that there exists a subset of about 20 carbon sources which have a significantly higher syntrophic potential than the average, and most syntrophies involving metabolisms viable on peripheral primary carbon sources also involve a carbon source from the core. In fact, there is only one notable exception to this core-periphery distinction— D-Alanine— which produces syntrophy as often with peripheral nodes as members of the core but produces syntrophy less often with the core than other members of the core.

We repeat this network analysis for 1) size-reduced metabolisms complemented by a full set of transport reactions, and 2) *E. coli*-sized metabolisms. The results are shown in Fig. S10 and demonstrate the robustness of the disassortative structure.

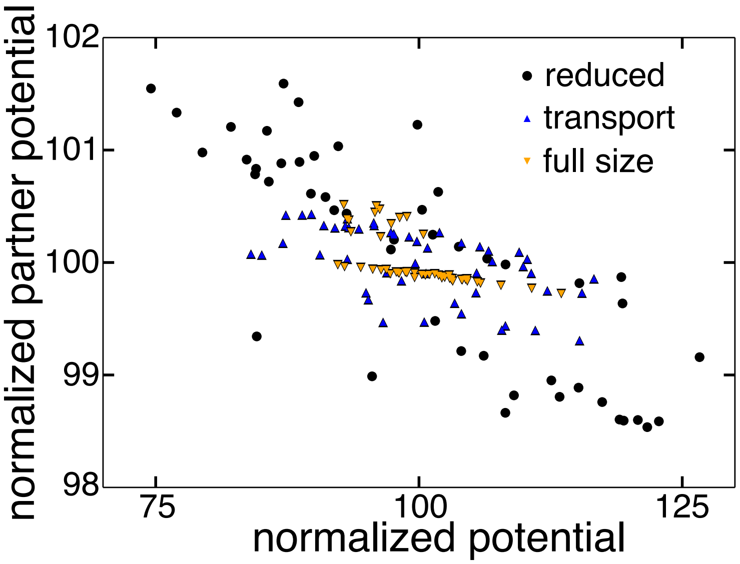

Figure S10: **Disassortativity of syntrophy across carbon sources using different constraints and metabolism sizes.** Comparison of the data shown in Fig. S9 and analogous data for 1) size-reduced metabolisms with a full set of transport reactions, and 2) *E. coli*-sized metabolisms. All networks feature a disassortative structure, as demonstrated by the statistical associations of -0.74 for size-reduced metabolisms, -0.57 for reduced metabolisms with transport reactions, and -0.65 for *E. coli*-sized metabolisms. We note that the correlations are even more pronounced if we account for the total number of additional carbon sources that pairs of metabolisms grow on due to syntrophy, rather than simply treating syntrophies as present or absent, as in our definition of pair syntrophic potential.

D-Alanine and the bias in carbon source syntrophy

Syntrophies yielding viability on D-Alanine (44.83 % of all syntrophic interactions) were over three times more common compared to the next most frequent carbon source, i.e., acetate (14.88%). In this section we explore the bias in carbon source syntrophy and what contributes to D-Alanine being the most common carbon source that can be metabolized through syntrophy.

One possible reason for the bias in carbon source syntrophy is that different numbers of reactions are required to metabolize different carbon sources. So carbon sources like D-Alanine may require on average far fewer reactions to metabolize than other carbon sources. To explore this possibility, we consider each carbon source and the set of all metabolisms able to metabolize it. For each metabolism, we systematically remove single reactions and test for viability on the primary carbon source. This provides a measure of the number of reactions required to metabolize a carbon source. The results of this computation are shown in Figure S11. We do not find any statistically significant relationship between the number of reactions needed to metabolize a carbon source and how often the carbon source is the subject of a syntrophy (see Figure S11). For example, of the carbon sources that can be metabolized via syntrophy Inosine requires the fewest essential reactions (360.35) but is less than half as often the subject of a syntrophy (4,156 vs 9,796) as D-Gluconate, which requires the most essential reactions (369.5).

While no link is apparent between the number of essential reactions associated with a carbon source and its frequency of being metabolized via syntrophy, the number of essential reactions may be too crude a metric to indicate the potential for syntrophic interactions. If we consider our population of metabolisms, we can order essential reactions into three categories. The first comprises core reactions (≈ 150) which every metabolism in our population possesses. These reactions are required in all metabolisms and all chemical environments to create biomass precursors. The second comprises reactions that all metabolisms viable on the same primary carbon source share. For carbon source C_i, we call these shared reactions the "common set of C_i". They include the reactions that are involved in importing the carbon source into the cell and thus allow for it to be used as a resource. Typically, the common set for a carbon source comprises ≈ 20 reactions. The third category comprises essential reactions specific to any one metabolism. Removal of any one of these ≈ 210 reactions from a metabolism abolish its viability. However, these reactions may not be essential (and may not even be present) in other metabolisms viable on the same carbon source.

Of the three categories of essential reactions, we focus on the common set of reactions for a carbon source, i.e. those reactions found in all metabolisms viable on that carbon source. As with the analysis of the average number of essential reactions, we do not find a correlation between how frequently a carbon source can be metabolized via syntrophy and the size of its common set (data not shown). However, we do find a relationship between

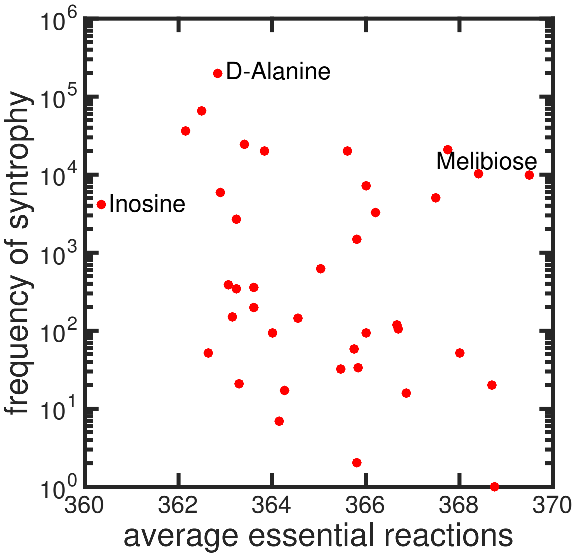

Figure S11: **The frequency of syntrophy versus the number of essential reactions**. The average number of essential reactions quantifies the number of reactions that when removed prevent viability on a primary carbon source. We find that there is no significant correlation between the average number of essential reactions and how often a carbon source is the subject of a syntrophy. As an example, D-Alanine is most frequently involved in syntrophic interactions, and yet it is associated with more essential reactions than multiple other carbon sources. Another example is Melibiose, which is associated with the most essential reactions but can be metabolized via syntrophy more often than Inosine, which requires the fewest.

how frequently a carbon source can be metabolized via syntrophy and how many pairs of metabolisms contain its common set of reactions. For each carbon source, we compute how often pairs of metabolisms that can syntrophically metabolize it also harbor its entire common set of reactions (see Figure S12). We find a correlation of 0.50 that is statistically significant ($p < .001$, using a t-test). In addition, 51.47% of pairs of metabolisms have all of the reactions for the common set of D-Alanine, a percentage that is greater than for all other other carbon sources except for D-Glucose (96.41%).

The results of Figure S12 indicate that the bias in how often carbon sources can be metabolized syntrophically may be related to how often pairs of metabolisms happen to contain the common set of reactions for different carbon sources; though it does not account for why the common set of reactions for some carbon sources, like D-Alanine, are more often present in pairs of metabolisms.

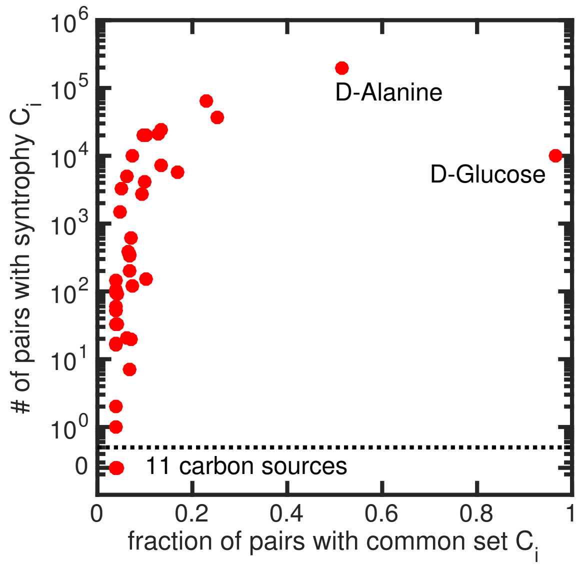

Figure S12: **The frequency of syntrophy involving a carbon source is associated with how often pairs of metabolisms harbor its common set of reactions**. Each red circle represents a primary carbon source. The horizontal axis depicts the fraction of all pairs of metabolisms that have the common set of reactions for that carbon source, while the vertical axis shows the number of metabolism pairs that can metabolize the carbon source syntrophically. We find a correlation of 0.50 that is statistically significant ($p < .001$, using a t-test).

The role of carbon source transport

The analysis in the previous section focused on what reactions were present in successful syntrophies. Here, we consider the inverse issue: what reactions are missing when pairs of metabolisms cannot syntrophically metabolize a particular carbon source. For each primary carbon source, we consider all pairs of metabolisms that cannot metabolize it syntrophically and compute which reactions from the common set[2] are most often missing from these pairs. We find that for 47 of the 50 primary carbon sources, the most often missing reaction from the common set is involved in carbon source transport, enabling a cell to import a carbon source from the external environment[3]. Interestingly, for D-Glucose and D-Alanine only 2.81% and 30.89%, respectively, of pairs lack this reaction, while for the other carbon sources on average 91.19% of pairs lack an essential transport reaction.

[2]See previous section for description of the common set of reactions for a carbon source.

[3]Metabolic flux balance analysis models often use different compartments corresponding to the external environment and inside the cell.
